# *Sasa Quelpaertensis Nakai* Induced Antidepressant-Like Effect in Ovariectomized Rats

**DOI:** 10.1155/2019/5815604

**Published:** 2019-07-11

**Authors:** Noof Abdullah Shaif, Donghyun Cho, Daehyuk Jang, Hyung Min Kim, Jin-Oh Chung, Sunmi Kim, Dae Bang Seo, Kyu-Ri Kim, Jaekyoon Shin, Insop Shim

**Affiliations:** ^1^Department of Science in Korean Medicine, College of Korean Medicine, Kyung Hee University, Seoul, 02447, Republic of Korea; ^2^Vital Beautie Research Division, AMOREPACIFIC R&D Unit, Gyeonggi-do 17074, Republic of Korea; ^3^Department of East West Medical Science, Graduate School of East-West Medical Science, Kyung Hee University, Kyunggi-do 17104, Republic of Korea; ^4^Department of Molecular Cell Biology, Sungkyunkwan University School of Medicine, Suwon, Republic of Korea; ^5^Department of Physiology, College of Medicine, Kyung Hee University, Seoul 02447, Republic of Korea

## Abstract

**Background:**

* Sasa quelpaertensis Nakai* extract (SQE) or dwarf bamboo has been extensively investigated for its antioxidant and anti-inflammatory effects; however, no previous study assessed its effect as an antidepressant agent. Therefore, this study was designed to examine the effect of oral SQE administration in ameliorating menopausal depressive symptoms and to evaluate its mechanisms in ovariectomized rats with repeated stress.

**Methods:**

All experimental groups except normal group underwent ovariectomy and then immobilization for 14 consecutive days. During these 2 weeks, two rat groups received SQE (100 and 300 mg/kg orally) and their cutaneous body temperature was measured. The tail suspension test (TST) and forced swim test (FST) were performed in order to evaluate depression-like behavior. Additionally, enzyme-linked immunosorbent assay (ELISA) and immunohistochemistry were carried out to evaluate the central monoaminergic neurotransmitter levels and activity.

**Results:**

Oral SQE (100 mg/kg) administration had reduced immobility time in TST and FST. Additionally, the SQE 100 and 300 mg/kg administration had decreased the cutaneous body temperature in the rats compared to those without treatment. In ELISA analysis, the SQE 100 group expressed elevated levels of serotonin and dopamine in the hypothalamus, prefrontal cortex, and hippocampus. Antityrosine hydroxylase (anti-TH) antibodies showed a tremendous increase in the density of TH positive cells in the locus coeruleus (LC) region of the SQE 100 group. Likewise, the SQE 100 elevated the number of tryptophan hydroxylase (TPH) and protein kinase C (PKC) immunoreactive cell counts and density in the hypothalamic region.

**Conclusion:**

These results suggested that the oral SQE administration induced the antidepressant-like effect in the ovariectomized rats with repeated stress via upregulating the levels of serotonin and dopamine through enhancing the expression of TH, TPH, and PKC in many brain areas.

## 1. Introduction

Major depressive disorders (MDD) are serious mood diseases that cause heavy economic and health burden since they affect 322 million people worldwide. Recently, the World Health Organization published a report indicating that the depressive disorders' prevalence is higher in women especially during the menopausal period due to the fluctuation of the sexual hormones levels [1].

Menopause is defined as a state of menstrual cycles' cessation in women for one year. Menopause could be as a result of a natural aging process, undergoing ovariectomy, or hysterectomy, or due to idiopathic premature ovarian failure [2–4].

For many years, several studies have attempted to elucidate the underlying pathology of menopausal depression through estrogen withdrawal theory. The menopausal period is characterized by fluctuation in estrogen hormones levels, which cause mood disturbances in the perimenopausal women [5]. Also, this theory suggested that reduced estrogen hormones influence brain neurotransmitters levels, such as serotonin, dopamine, and norepinephrine [4–6].

The menopausal women not only show depressive symptoms but also show hot flushes (HF), sleep disturbance, and bone fractures [6–8]. HF is the most bothersome symptom which is characterized by profuse sweating and face flushing. The mechanism of HF is still not clear; however, the authors [9] reported a potential correlation between HF and norepinephrine (NE) levels. Presence of abnormal NE levels in the brain, accompanied by low estrogen levels, is noticed to cause a dysfunction in the body temperature.

Several studies have addressed the neuroprotective and neuroregulatory role of estrogen hormones [10–13]. Estrogen regulates monoaminergic neurotransmitters levels like dopamine and serotonin [14, 15] and protects neurons by upregulating protein kinase C (PKC) activity and expression in the central nervous system [16]. PKC regulates the synaptic membrane and neuronal plasticity, releases neurotransmitter, and prevents neurons apoptosis [16–20]. It is renowned now that low estrogen hormones, as well as stress, attenuate hippocampal PKC expression, which is believed to be involved in the pathology of the depressive disorders [21–23].

There is increasing interest in alternative medicine and natural herbs that produce many beneficial and healing properties without any adverse effects.* Sasa quelpaertensis Nakai* extract (SQE) or dwarf bamboo (Korean name, Jeju-Joritdae) is a unique bamboo grass which belongs to the Poaceae family that is only growing in Halla Mountains on Jeju Island, South Korea. Its leaves are edible and are used as tea with proven antiulcerogenic, antiobesity, and anticancer effects [24–26].

Recently, this bamboo grass gained more attention in traditional medicine due to its dietary phytochemicals (including p-coumaric acid and tricin) that boost general health [27–32]. It is reported that bamboo leaf extract enhanced the levels of acetylcholine and *γ*-aminobutyric acid (GABA) in rat's hippocampus and improved the learning and memory ability of dementia model rats [33]. Additionally, the authors [34] addressed bamboo leaf extract to have an anxiolytic effect on the high-fat diet-induced anxiety in mice. However, no previous study reported the effect of bamboo leaf extract on the central monoaminergic neurotransmitters and its mechanisms in ameliorating depressive symptoms. Therefore, in this present study, we aimed to investigate the antidepressant-like effect of oral SQE and to elucidate its mechanisms in the ovariectomized and repeatedly stressed rats.

Previous studies have reported that the menopausal period in women's life is so stressful [35–37]. Therefore, we performed immobilization to induce stress in the ovariectomized rats in order to intensify the depression-like symptoms in our animal model.

## 2. Materials and Methods 

### 2.1. *Sasa quelpaertensis Nakai* Extract (SQE) Preparation

*Sasa quelpaertensis Nakai* leaves were plucked from Halla Mountain (Jeju Island, Korea) and dried for 24 hr at 60°C. The dried* Sasa quelpaertensis Nakai* leaves were firstly extracted with 50% ethanol for 2 hr at 70°C and filtered to obtain 1st supernatant. The remaining residues were extracted with water at 80°C for 2 hrs once more and filtered, to obtain 2nd supernatant. Then, two clear supernatants were mixed and concentrated in a vacuum evaporator, to obtain* Sasa quelpaertensis Nakai* concentrate, and stored at −20°C for future use.

### 2.2. Animal Experiment

Eight-week-old female Sprague-Dawley (SD) rats were used in two experiments and were obtained from Samtako Animal Company (Seoul, Korea). Rats were housed in their cages and kept under a standard degree of temperature and humidity with a light/dark cycle each day. The animals were allowed to acclimatize themselves for 1 week before the experiments began. Experiments were authorized by the Institutional Animal Care and Use Committee at Kyung Hee Medical Center.

To generate the menopausal animal model, ovariectomy surgery (OVX) was achieved by pentobarbital sodium (50 mg/kg, i.p.). According to the method described in this study [38], the surgical area was wiped with ethanol and 2 cm midline dorsal skin incision was made. Once the ovaries were recognized, they were removed and then the muscle and skin layer were stitched up separately by absorbable and nonabsorbable suture, respectively (Blue Nylon sutures- 4/0, AILEE Comp. Ltd., Korea). The surgical incision was cleaned with povidone-iodine and all rats were allowed 7 days for postsurgery recovery under similar laboratory conditions.

The first experiment was intended to examine the effect of SQE on behavioral tests and on upregulating neurotransmitter levels in the brain. The second experiment was designed to elucidate the SQE mechanisms that enhance neurotransmitter levels. Thirty-eight rats (in each experiment) were distributed randomly into four experimental groups: nonoperated and nonstressed group (normal,* n *=8), ovariectomized and stressed group (control,* n* =10), ovariectomized group which underwent stress with SQE 100 mg/kg being orally administered (SQE 100,* n* =10), or with SQE 300 mg/kg (SQE 300,* n* =10).

**Figure 1:**
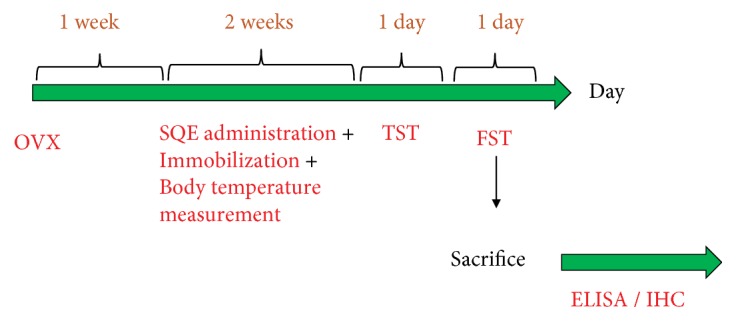
*Experimental schedule of developing ovariectomized rat model, repeated stress, and SQE administration*. OVX: ovariectomy, TST: tail suspension test, FST: forced swim test, ELISA: enzyme-linked immunosorbent assay, and IHC: immunohistochemistry.

SQE was administered orally by a rat gavage needle and was prepared freshly before every experiment. The SQE groups were treated with SQE (100 and 300 mg/kg) for 14 consecutive days; however, sterile saline was administered to the normal and control groups. All procedures of both experiments were conducted in the same manner; nevertheless, the extraction process of rats' brains was changed based on the designed tests (Figure 1) [39].

### 2.3. Stress Procedure

After the postsurgical recovery period, the ovariectomized rats underwent immobilization according to the chronic stress protocol [40–42]. The stress was conducted via plastic bags (a disposable rodent restraint cone, Harvard Instrument, USA) for 2 hr daily for 14 consecutive days. All groups, except normal group, underwent the same stress.

One day after the end of immobilization, behavioral tests were conducted; then rats were sacrificed and brain tissues were immediately collected and stored at −80°C.

### 2.4. Peripheral Body Temperature Measurement

The proximal part of the rat's tail skin temperature was assessed during the treatment period. We utilized an Infrared Thermometer for rodents (153-IRB, Braintree Scientific, USA) [39, 43].

### 2.5. Behavioral Tests

#### 2.5.1. Tail Suspension Test (TST)

The rats were suspended using an adhesive tape around its proximal part of the tail which was fixed to a metal hook located at the top of each compartment in a wooden box (width: 30 cm, height: 54 cm, and diameter: 47 cm). Rats were taped for 6 min to calculate the immobility time manually [44, 45].

#### 2.5.2. Forced Swim Test (FST)

After 2 weeks of treatment, rats were acclimated for 15 min before the test by placing them in a transparent Plexiglas cylinder (50 cm × 20 cm diameter) filled with water. After 1 day, all rats underwent a forced swim test for 6 min. The immobility behavior was defined as the time of absence of rat movement while floating on water [46].

### 2.6. ELISA Analysis for Serotonin (5-HT) and Dopamine (DA) in the Prefrontal Cortex, Hippocampus, and Hypothalamus

After behavioral testing, the animals of the 1st experiment were deeply anesthetized with sodium pentobarbital (80 mg/kg, administered i.p.) and the brains were immediately removed and coronally sectioned by using rodent brain matrix (ASI instruments Inc., MI, USA). The prefrontal cortex, hippocampus, and hypothalamic regions of the brain were punched out on a cold plate and stored at −80°C until the assay.

The brain tissues were standardized with a lysis buffer (137 mM NaCl, 20 mM Tris, 10% glycerol, 1% NP40, 1 mM PMSF, 1 mg/ml leupeptin, 10 mg/mL aprotinin, and 0.5 mM sodium vanadate) and then incubated at 4°C. The serotonin and dopamine levels in the 3 previously mentioned areas were evaluated by Serotonin/Dopamine ELISA kit (Catalog number: BA E-5900 and BA E-5300, LDN, Germany).

### 2.7. Immunohistochemistry Analysis for Tryptophan Hydroxylase (TPH), Tyrosine Hydroxylase (TH), and Protein Kinase C (PKC)

After sacrificing the rats of the 2nd experiment, the brains were postfixed and cryoprotected with sucrose 20% in PBS solution at 4°C. Coronal sections (30 *μ*m) were collected from the locus coeruleus (LC) and hypothalamic regions. The sections were immunostained for TH expression (primary mouse anti-TH antibody [1:1000]; TH [45]: sc-136100, Santa Cruz Biotechnology, USA), TPH expression (primary sheep anti-TPH antibody [1:500]; AB1541, Germany), and PKC expression (primary mouse anti-PKC antibody [1:500]; anti-PKC antibody [MC5] (ab31), Abcam, United Kingdom). After 72 h, the sections were incubated with secondary antibodies (1:200 dilution, Vector Laboratories Co., USA).

The sections were kept in ABC reagent (Vectastain ABC HRP kit, Vector Labs. Co., USA) and then in 3,3′-Diaminobenzidine (DAB; Sigma-Aldrich Chemical Co., Germany) for 1 min. All tissues were washed and fixed onto slides and then by an Olympus BX53 microscope (Olympus Scientific Solutions Americas Corp., USA) images were obtained and processed.

The immunopositive cells in the LC and hypothalamic region were evaluated at 40x, 100x, and 200x magnification and the counting was performed within a square (150 × 150 *μ*m^2^), in which the LC was localized in Bregma -9.72 mm, Interaural -0.72 mm and the hypothalamus was in Bregma -2.40 mm, Interaural 6.60 mm in accordance with the 5th edition of rat brain atlas of Paxinos and Watson. TH, TPH, and PKC-immunopositive cells were recognized by brown or violet spots in the LC and hypothalamus, respectively. All immunohistochemical data were independently observed and analyzed by two blind researchers.

### 2.8. Statistical Analysis

The results of this study are expressed as means ± standard error of the mean (SEM) by using SPSS 23.0 software (SPSS Inc., USA). One-way ANOVA with subsequent post hoc LSD test was conducted in which *P* values of ≤ 0.05 were counted as statistically significant.

## 3. Results

### 3.1. Effect of Oral SQE Administration on TST

**Figure 2:**
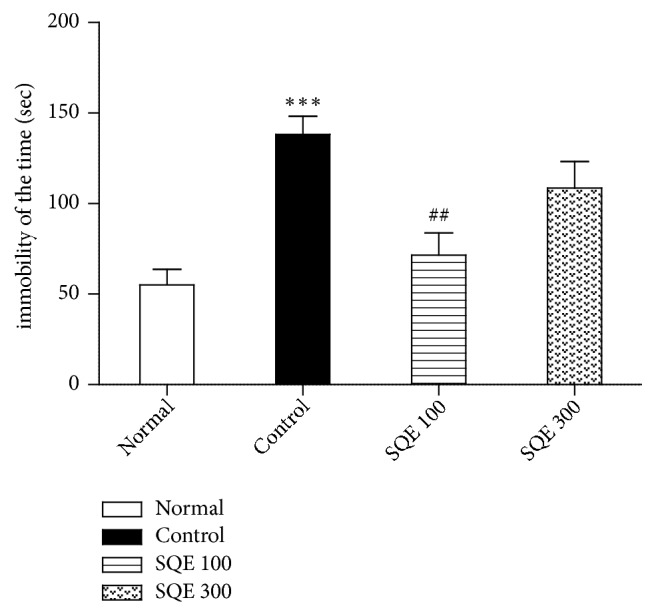
*Effect of oral SQE on time remaining immobile in the tail suspension test (TST)*. Values represent mean ± SEM. *∗∗∗ p* <0.001 compared to normal group while ##* p* <0.01 compared to control group. The* p* value of the TST test was < 0.001.

The ovariectomized rats who were subjected to stress for 2 weeks (control group) displayed depression-like behavior, characterized by remaining immobile for a longer time as compared to the normal group (*p* <0.001). However, SQE 100 mg/kg administered group significantly reduced immobility time during 6 min in the TST, in comparison with those in control group [F (3, 36) = 9.767,* p* <0.001, Figure 2].

### 3.2. Effect of Oral SQE Administration on FST

**Figure 3:**
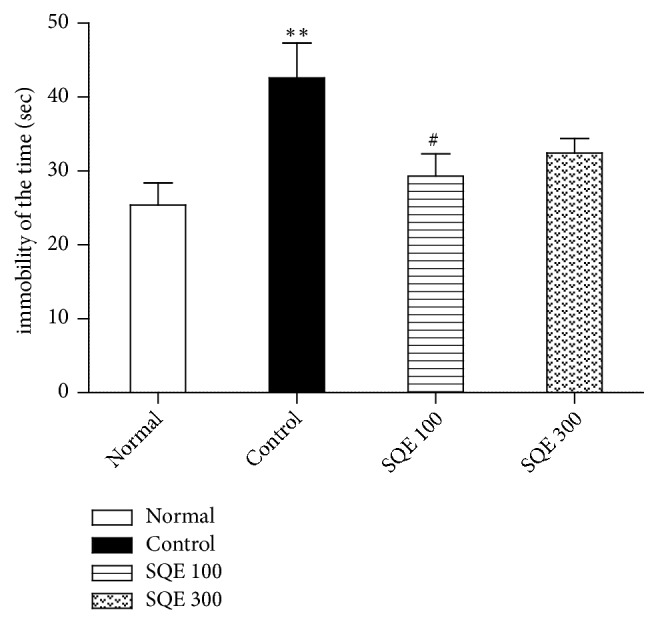
*Effect of SQE administration on immobility time in the forced swim test (FST)*. Values are as mean ± SEM. *∗∗ p* <0.01 vs. normal group and #* p* <0.05 vs. control group.

ANOVA revealed a significant reduction in the immobility time of FST in SQE 100 administered group compared to the control value [F (3, 36) = 4.608,* p* <0.01, Figure 3]. However, the SQE 300 group showed no significance in both TST and FST.

### 3.3. Effect of SQE Administration on Peripheral Body Temperature

**Figure 4:**
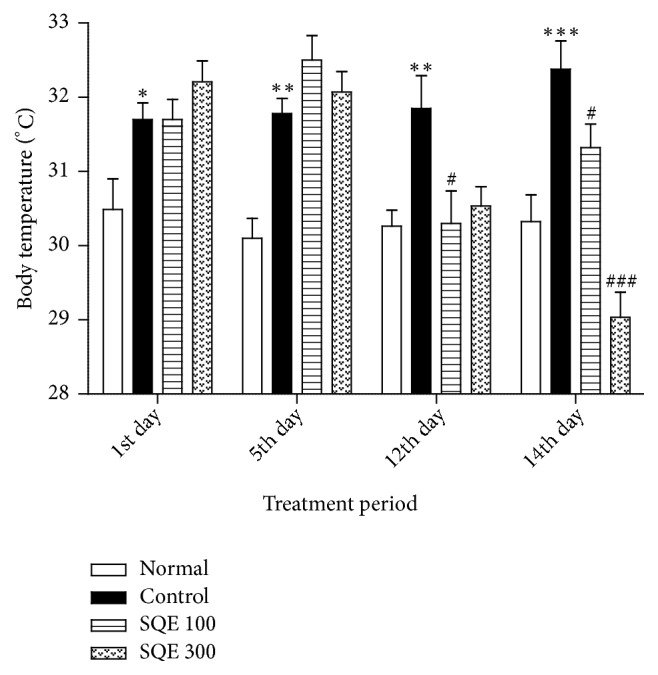
*Effect of oral SQE administration on the peripheral body temperature of rats with ovariectomy*. *∗ p* <0.05, *∗∗ p* <0.01, and *∗∗∗ p* <0.001 compared to normal group. #* p* <0.05 and ###* p* <0.001 compared to control group. By one-way ANOVA, the* p* value of the test was < 0.001.

After one week following ovariectomy, rats in the control group displayed a gradual increase in the cutaneous body temperature compared to those in the normal group (*p* <0.001). On the other hand, the SQE 100 and 300 groups exhibited a significant decrease in the body temperature in the last day of treatment period [F (3, 37) = 27.524,* p* <0.001, Figure 4].

### 3.4. Effect of Oral SQE on 5-HT and DA Levels in the Prefrontal Cortex (PFC), Hypothalamus, and Hippocampus

**Figure 5:**
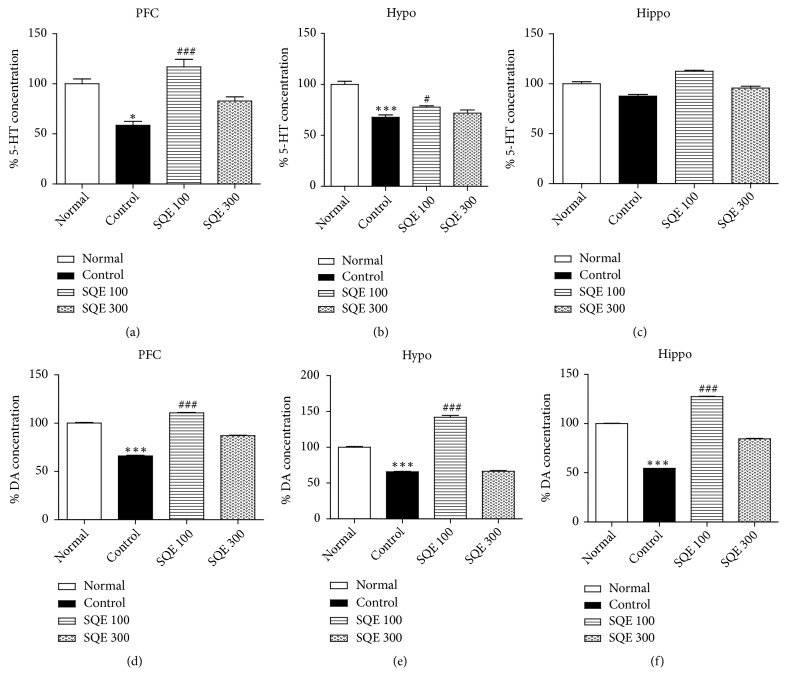
*Effect of SQE administration on serotonin (5-HT) and dopamine (DA) levels in the brain of the ovariectomized rats*. (a-c) 5-HT levels in the prefrontal cortex, hypothalamus, and hippocampus after SQE 100 and 300 mg/kg administration. (d-f) DA levels in the SQE 100 and 300 groups in the prefrontal cortex, hypothalamus, and hippocampus. *∗ p* <0.05 and *∗∗∗ p* <0.001 vs. normal group, while #* p* <0.05 and ###* p* <0.001 vs. control group. The* p* value of the ELISA test was < 0.001.

ELISA analysis presented that 100 mg/kg of SQE administration for 2 weeks markedly upregulated the level of 5-HT in the PFC and hypothalamus, and DA in all areas compared with the control group [(*n* =6), F (3, 24) = 16.824, F (3, 24) = 3.932, F (3, 21) = 6.510, F (3, 21) = 16.323, F (3, 22) = 11.787,* p* <0.001, Figures 5(a)–5(f)]. However, the SQE 300 group produced no significant increase in the total concentration of 5-HT or DA levels compared to the control group.

### 3.5. Effect of Oral SQE on TH Immunohistochemistry Reaction in Locus Coeruleus (LC)

**Figure 6:**
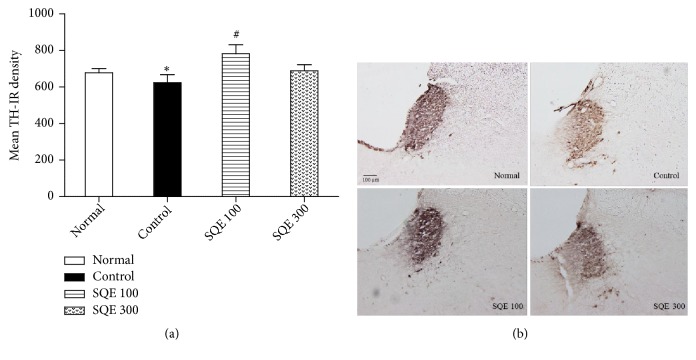
*Effect of oral SQE on tyrosine hydroxylase (TH) immunopositive cells in the locus coeruleus (LC)*. (a) Mean TH-immunopositive cell density in the LC region for all experimental groups. *∗ p* <0.05 vs. normal group, while #* p* <0.05 vs. control group. (b) Representative images of TH-IR cells expression in the LC region with 100x magnification.

The expression of TH positive cells in the control group was significantly reduced compared with that in the normal group (*p* <0.05). The diminished expression of TH-immunoreactivity (TH-IR) in the control group was notably restored in the SQE 100 group [(*n* =6), F (3, 21) = 3.309,* p* < 0.001, Figure 6(a)]. The representative images of TH-IR cells per group from the LC area are shown in Figure 6(b).

### 3.6. Effect of SQE Administration on TPH Expression in the Hypothalamic Nuclei

**Figure 7:**
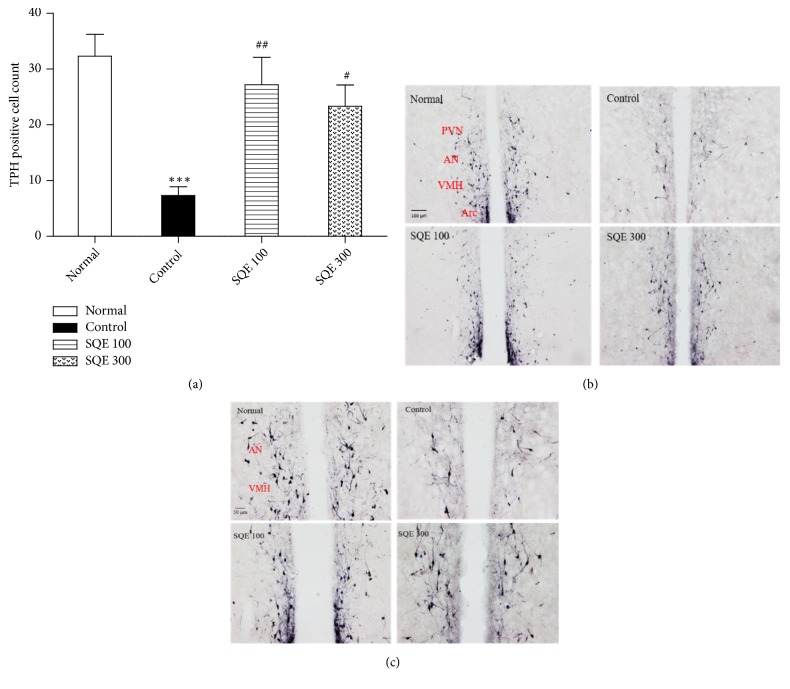
*Effects of oral SQE on the hypothalamic tryptophan hydroxylase (TPH) expression*. (a) The TPH+ cells count in both SQE treated groups compared to the control group. *∗∗∗ p* <0.001 compared to normal group, while #* p* <0.05 and ##* p* <0.01compared to control group. The* p* value was <0.001 by one-way ANOVA. (b, c) Representative images of TPH+ cells in all groups using anti-TPH immunohistochemistry in the hypothalamic region (the scale bars were 100 and 50 *μ*m). PVN: paraventricular hypothalamic nucleus, AN: anterior hypothalamic nucleus, VMH: ventromedial hypothalamic nucleus, Arc: arcuate nucleus.

The hypothalamic TPH+ cells in the control group were drastically diminished in comparison with the normal group (*p* < 0.001). However, both SQE 100 and 300 groups significantly increased the TPH-IR cells (*p* < 0.01) and (*p* < 0.05) [(*n* =6), F (3, 23) = 9.136, Figure 7(a)]. TPH+ cells were apparent with high cells number in the SQE groups' tissues in the anterior hypothalamic nucleus (AN), ventromedial hypothalamic nucleus (VMH), paraventricular hypothalamic nucleus (PVN), and arcuate nucleus (Arc) (Figures 7(b) and 7(c)).

### 3.7. Effect of Oral SQE on Immunohistochemistry Reaction of TPH in the Peduncular Part of the Lateral Hypothalamus (PLH)

**Figure 8:**
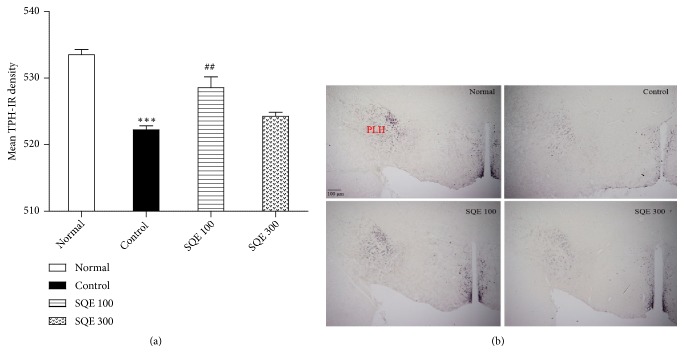
*Effect of SQE administration on TPH activity in the peduncular part of the lateral hypothalamus (PLH)*. (a) TPH+ cell density in the PLH in the SQE 100 and 300 groups compared to the control group. *∗∗∗ p* <0.001 vs. normal group, while ##* p* <0.01 vs. control group. (b) Representative images of TPH-IR cell expression in the lateral hypothalamic peduncular region of all groups with 40x magnification.

In PLH, the expression of TPH-IR cells in the control group was radically diminished as compared with the normal group (*p* <0.001). The diminished TPH expression in the rats with no treatment was considerably enhanced in the SQE 100 group [(*n* =6), F (3, 22) = 3.975,* p* < 0.01, Figure 8(a)]. This enhanced expression in the SQE 100 group's tissue is shown in Figure 8(b).

### 3.8. Effect of SQE Administration on PKC Expression in the Hypothalamic Area

**Figure 9:**
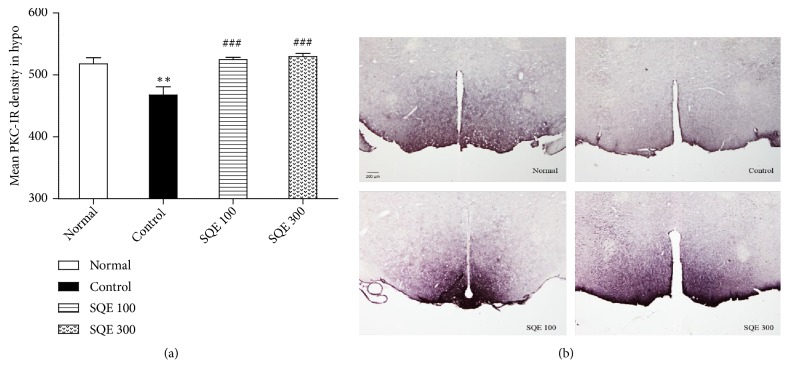
*Effect of oral SQE on protein kinase C (PKC) activity in the hypothalamic area*. (a) Mean PKC+ cell density in both SQE groups compared to the control group. *∗∗ p* <0.01 vs. normal group and ###* p* <0.001 vs. control group. (b) Representative images of PKC+ cells activity in the hypothalamus of all groups in which the scale bar denotes 200 *μ*m.

In the hypothalamus of the control group, the expression of PKC-IR cells was remarkably attenuated in comparison to the normal group (*P*<0.01). On the other hand, the SQE treated groups significantly enhanced the expression of PKC+ cells in both SQE 100 and 300 group (*p* < 0.001) [(*n* =6), F (3, 23) = 13.558, Figure 9(a)]. This intense PKC+ cell expression in both SQE groups' tissues is shown in Figure 9(b).

## 4. Discussion

Many literature reviews have reported that the SQE has antioxidant and anti-inflammatory effects and produces an antifatigue effect on locomotor activity in the mice [27–30, 33, 47]; however, no previous study presented the effect of oral SQE administration on neurotransmitters or as an antidepressant drug in the menopausal animal model. Therefore, this study was designed to investigate the effect of oral SQE on the ovariectomized rats with repeated stress and to explore its mechanisms that exhibited the antidepressant-like effect. We intended to administer two different doses of SQE (100 and 300 mg/kg) since SQE chemical composition and bioavailability are well studied in these two doses [28].

Our present data demonstrated that continuous administration of the SQE orally for 2 weeks induced an antidepressant-like effect on the ovariectomized rat with repeated stress. During behavioral tests (TST and FST), a significant increase in the immobility time was noticed in the control group as compared to the normal group. However, only the SQE 100 group reduced immobility time in both TST and FST. The antidepressant-like effect of the SQE 100 group was probably as a result of neurotransmitter regulation in many brain areas. It is known that further increases in the dosage lead to increases in effectiveness; however, in case of the SQE 300 group, the higher dose did not improve depressive symptoms. The exact mechanism warrants further study.

Previous studies addressed that low estrogen hormones, accompanied by abnormal NE levels, cause changes in core body temperature, which lead to hot flushes (HF). HF occurs due to a dysfunction in the noradrenergic system, which in sequence affects the core and cutaneous blood circulation [9]. Following ovariectomy by one week and during the immobilization period, a gradual elevation in the peripheral body temperature was observed in the rats of the control group [48]. On the other hand, the SQE groups exhibited a notable gradual decrease in body temperature by ~2-3°C which was distinct on the last day of the treatment. Though the exact mechanism is not clear yet, the reduction in the body temperature may appear as a result of the serotonin and dopamine levels upregulation since these both monoamines are involved in the thermoregulation [49–51].

In our study, we did not observe more long-term effects of SQE on TST/FST and body temperature. There may be a dissociating effect of SQE on immobility and body temperature between low and high doses. A low dose of SQE has a more depressive-like action, whereas a higher dose has a more reducing effect on peripheral body temperature.

Several researches have pointed to the relationship between the serotonin and dopamine levels and brain functions [52, 53]. To investigate the changes which occurred in the neurotransmitter levels, we performed ELISA tests on three brain areas (hypothalamus, PFC, and hippocampus) to assess the serotonin and dopamine levels after SQE 100 and 300 mg/kg administration. We noticed that the level of 5-HT (in the hypothalamus and prefrontal cortex) and DA (in the hypothalamus, hippocampus, and prefrontal cortex) was significantly upregulated in the SQE 100 treated group. However, the SQE 300 group showed no significant elevation in the neurotransmitter levels in all of the three previously mentioned brain areas. This may explain the nonsignificant effect of the SQE 300 in both TST and FST.

Therefore, in order to elucidate the SQE mechanisms that upregulated neurotransmitter levels, immunohistochemistry was conducted using antityrosine hydroxylase (TH) in the locus coeruleus (LC). Tyrosine hydroxylase is the enzyme that produces L-dopa, for the synthesis of dopamine and norepinephrine. Recent reports indicated that locus coeruleus contains tyrosine hydroxylase positive cells (LC-TH+) that cause dopamine release in many brain areas. Moreover, either a diminution of LC-TH immunoreactive cells expression or a blockading of the norepinephrine transporter in the LC area suppresses the DA release, which indicates that LC neurons can release DA and NE [54–58]. In concordance with this study [59], our data also displayed that stress is affecting the neurons in the LC (as in control group) and showed the diminished density of TH+ cells in the LC region; conversely, the SQE 100 group notably increased the TH-IR cells density in the LC. Enhanced TH activity could elucidate the increase observed in dopamine levels within several brain areas.

It is well known that a functional abnormality of monoamine including 5-HT, DA, and NE in the hypothalamus plays a role in the pathogenesis of certain types of depression. Recent studies suggest that the onset of depression is accompanied by the hypothalamus-pituitary-adrenal axis hyperfunction [60–62]. In particular, estrogen is known to increase serotonergic activity by stimulating serotonin synthesis and transmission and downregulating degradation of 5-HT in the brain [7, 63]. Therefore, since tryptophan hydroxylase is the enzyme responsible for the synthesis of serotonin, a TPH immunohistochemistry was carried out in the hypothalamic area to clarify the SQE mechanism that induced an elevation in the serotonin levels. The rats in control group exhibited a lower TPH-IR cells number in the hypothalamic nuclei as in this study [64], while the SQE 100 and 300 administration enhanced these cells significantly in the anterior hypothalamic nucleus (AN), ventromedial hypothalamic nucleus (VMH), paraventricular hypothalamic nucleus (PVN), and arcuate nucleus (Arc).

Furthermore, TPH+ cell densities in the lateral hypothalamic peduncle (PLH) in the control group displayed a hypodensity as compared to the normal group. This low density in the control group was considerably increased in the SQE 100 treated group. Upregulation of TPH activity in the hypothalamus after oral SQE administration could explain the elevation of the serotonin levels that occurred in the ovariectomized rats' brains [65, 66]. All previously displayed data indicate that the increases in TH and TPH activity were eventually accompanied by an elevation in the levels of serotonin and dopamine.

The relationship between diminished protein kinase C (PKC) activity and the depressive disorders has been addressed in many studies [21–23]. Also, the link between low TH and TPH activity and low PKC expression has been indicated in many researches [67–69]. Therefore, immunohistochemistry for PKC was conducted in the hypothalamic region. In the control group, PKC+ cells exhibited diminished density in comparison with the normal group. The lower PKC+ cell density in the hypothalamic area was drastically enhanced in both SQE 100 and 300 treated groups.

Taken together, all findings explain that oral SQE administration induced the antidepressant-like effect on the ovariectomized rats with repeated stress via upregulation of the serotonin and dopamine levels through enhancing the expression of tyrosine hydroxylase, tryptophan hydroxylase, and protein kinase C in the brains of menopausal depression model.

## Data Availability

The data used to support the findings of this study are available from the corresponding author upon request.
